# Brain Magnetic Resonance Imaging Findings in Infantile Spasms

**DOI:** 10.3390/neurolint14010021

**Published:** 2022-03-03

**Authors:** Osama Y. Muthaffar

**Affiliations:** Section of Neurology, Department of Pediatrics, Faculty of Medicine, King Abdulaziz University, Jeddah 21589, Saudi Arabia; oymuthaffar@kau.edu.sa

**Keywords:** epilepsy, infantile spasms, hypsarrhythmia, brain MRI

## Abstract

Background: Infantile spasms are an age-specific epileptic disorder. They occur in infancy and early childhood. They can be caused by multiple etiologies. Structural abnormalities represent an important cause of infantile spasms. Brain magnetic resonance imaging (MRI) is one of the integral modalities in the evaluation of this condition. Purpose: The aim of this study is to review and analyze the clinical characteristics and brain MRI findings in a cohort of children diagnosed with infantile spasms. Material and Methods: A cohort of fifty-six children diagnosed with infantile spasms in infancy and early childhood was included. All of them underwent brain MRI for evaluation. The study was conducted in the period from January 2016 to January 2020. Results: Females comprised 57% of the cohort. The mean age for seizure onset was 5.9 months (SD 2.7). Forty-one patients (73%) had active epilepsy, and 51% were diagnosed with global developmental delay. Consanguinity was present in 59% of the cohort. Most of the follow-up MRIs showed structural abnormalities (84%). Hypoxia was reported in 17% of MRIs. Malformations of cortical development were seen in five patients. Brain MRI findings were normal in 16% of patients, and delayed myelination was seen in nineteen patients. Most of the children with active epilepsy (64%) and developmental delay (82%) had an abnormal brain MRI. It was noticed that abnormal second brain MRIs were more likely to be associated with active epilepsy and developmental delay (*p* = 0.05). Conclusions: Brain MRI is an integral part of infantile spasms’ clinical evaluation. Infantile spasms and abnormal brain MRI can be associated with active epilepsy and global developmental delay.

## 1. Introduction

Infantile spasms (IS) are one of the early-onset epilepsy syndromes. They are characterized by the occurrence of flexion, extension or a mixed cluster of spasms. They can occur up to tens of times per day. Sometimes, they can be associated with sudden crying, eye closure or apnea [1]. Usually, they occur in infancy before the age of 1 year and, to a lesser extent, in early childhood years. They can lead to developmental regression. Infantile spasms constitute 2–4% of childhood epilepsies [2,3]. The International League Against Epilepsy (ILAE) designated West syndrome as an electroclinical syndrome with onset in infancy and epileptic spasms as a type of seizure. It was first described by Dr. W J West in a letter to the editor of The Lancet in 1841 describing IS in his own son [4].

The prognosis of infantile spasms is usually poor. Most children diagnosed with IS will develop intellectual delay; autistic features; and epilepsy syndromes, such as Lennox–Gastaut syndrome [5,6]. The symptomatic type of IS usually presents with delayed milestones before the onset of IS. Moreover, etiologies are usually found in 60–70% of children with symptomatic IS [7,8,9]. Patients with symptomatic infantile spasms will have a higher risk for the development of intellectual delay, autism and epilepsy compared with those with cryptogenic or idiopathic spasms [10,11].

Neuroimaging is an integral part of IS evaluation. Brain MRI should be conducted in all children diagnosed with IS. High-resolution 3 Tesla MRI brain scanning, which includes coronal and axial T2-weighted sequences, along with sagittal, axial and coronal T1-weighted sequences, should be considered in children with IS. It is also recommended to perform MRI brain scanning before commencing antiseizure medications (ASMs), which can sometimes cause imaging changes. A follow-up MRI brain scan is also advised, especially if the first one was performed before the age of 2 years, due to immature myelination patterns seen in infants [12,13,14].

Multiple neuroimaging patterns have been reported in the literature in association with infantile spasms. Some examples include hypoxic ischemic encephalopathy; malformations of cortical development; infections, such as meningitis; vascular insults; post-trauma; metabolic changes; and structural lesions, such as corpus callosum dysgenesis [15,16].

Few studies have reviewed the brain MRI abnormalities seen in IS. This study aims to retrospectively review and analyze the clinical characteristics and brain MRI findings in a cohort of children diagnosed with infantile spasms in a single center at King Abdulaziz University Hospital, Jeddah, Saudi Arabia.

## 2. Materials and Methods

### 2.1. Study Design

This is a retrospective review of children diagnosed with infantile spasms who underwent MRI brain scans. Fifty-six children diagnosed with IS were included. All of them underwent 3 Tesla MRI brain scans (Magnetom Trio, Siemens, Erlangen, Germany) for evaluation in the period from January 2016 to January 2020. The study was conducted at King Abdulaziz University Hospital, Jeddah, Saudi Arabia. MRIs were performed with sedation using chloral hydrate to minimize motion artifacts.

### 2.2. Patient Characteristics

Inclusion criteria were (1) infants and children diagnosed with infantile spasms and whose EEG showed findings compatible with hypsarrhythmia, and (2) infants and children who underwent 3 Tesla MRI brain scans at least twice during the course of their disease. Exclusion criteria were (1) an epilepsy diagnosis other than infantile spasms, and (2) the unavailability of MRI brain scans. The study was approved by the Research Ethical Board at the Faculty of Medicine at King Abdulaziz University Hospital, Jeddah, Saudi Arabia. 

### 2.3. Statistical Analysis

Data were entered into Statistical Package of Social Sciences version 22.0 (SPSS, Inc., Chicago, IL, USA). Qualitative data are described for all the study variables using frequency, number and percent. Quantitative data are described using mean, standard deviation and range (minimum and maximum). Chi-square test was used to compare categorical variables. A *p*-value of less than 0.05 was considered to be statistically significant. Logistic regression analyses were applied between MRI brain scans and multiple variables to evaluate their relationship. 

## 3. Results

**Figure 1:**
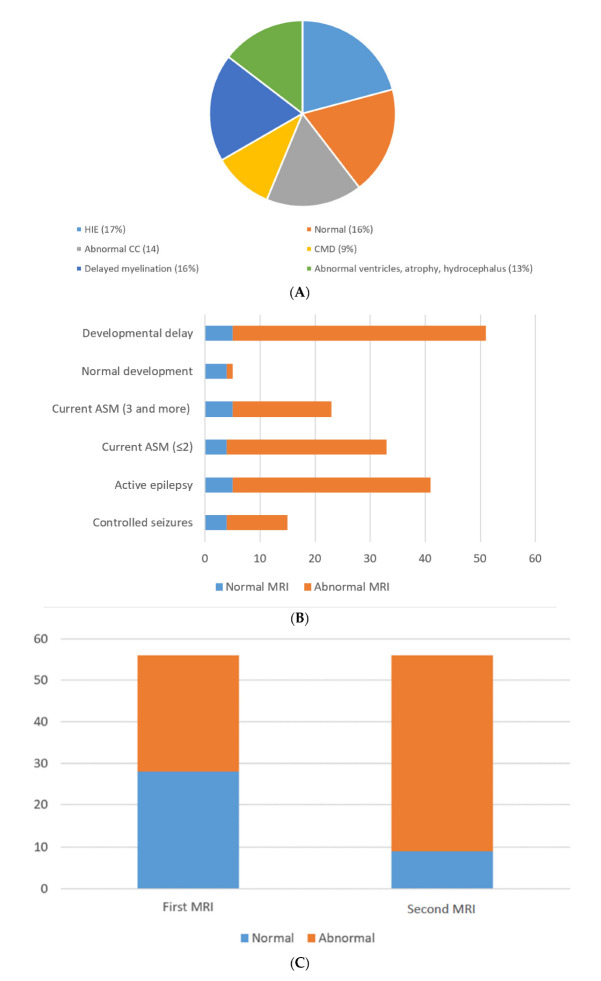
(**A**) MRI brain etiologies. (**B**) Normal and abnormal 2nd MRI compared to development, medications and epilepsy. (**C**) Normal and abnormal MRIs upon follow-up.

**Figure 2:**
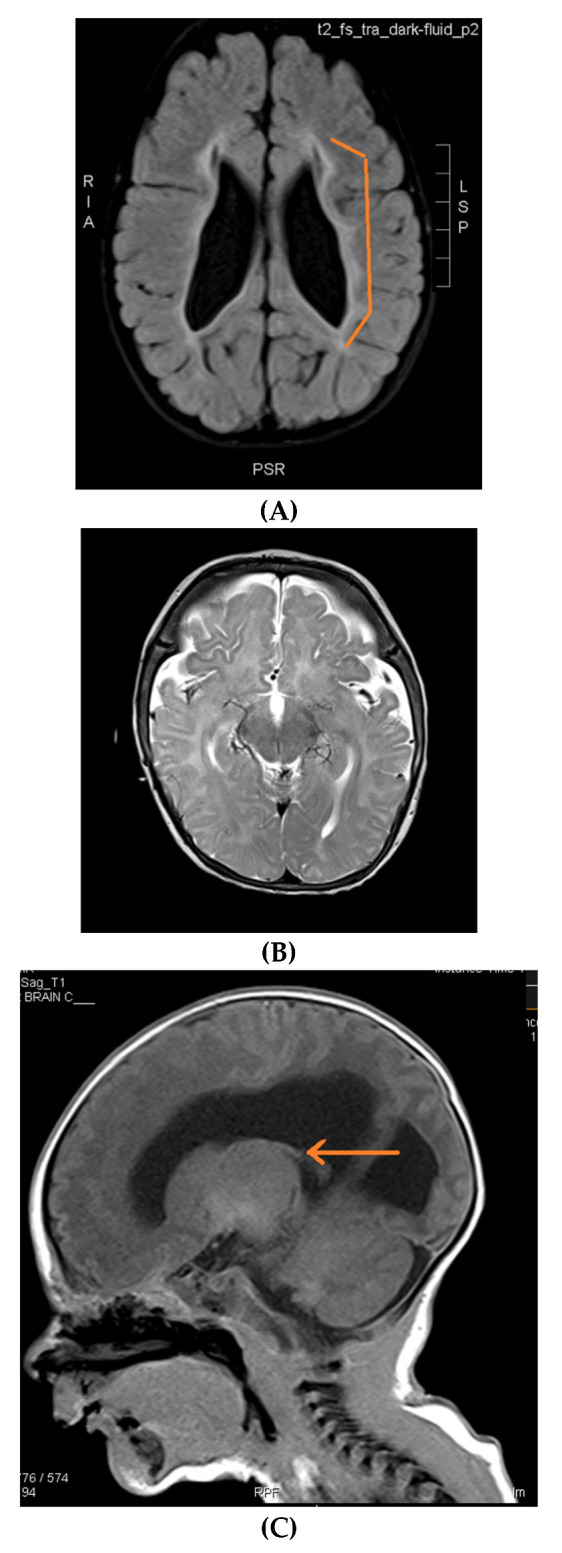

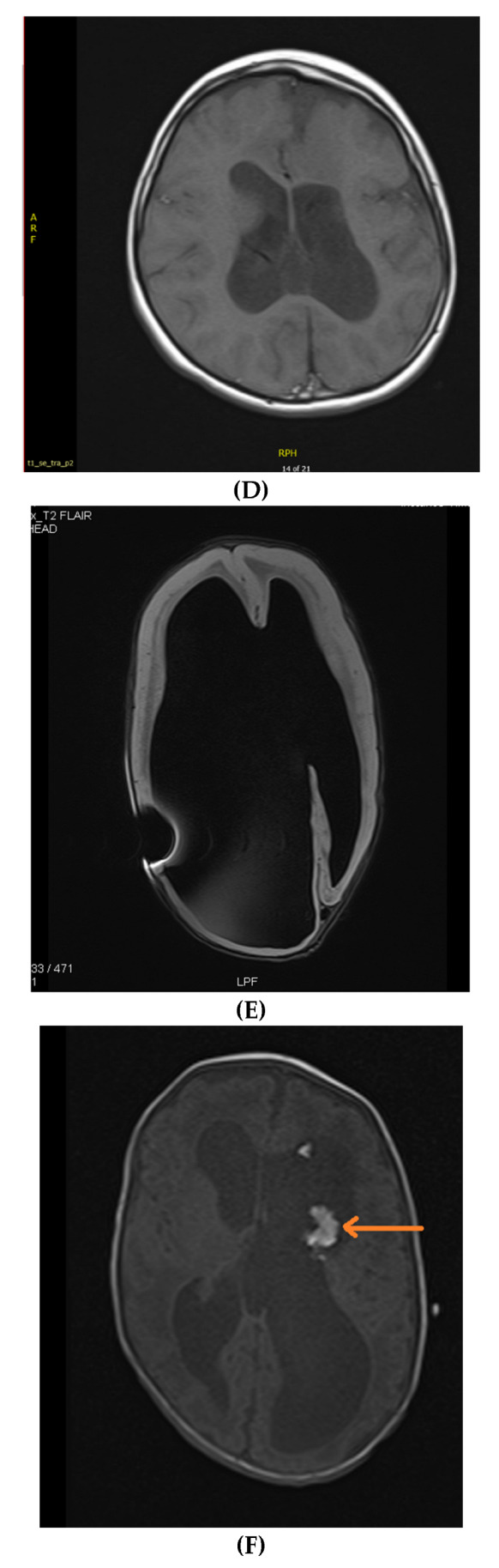

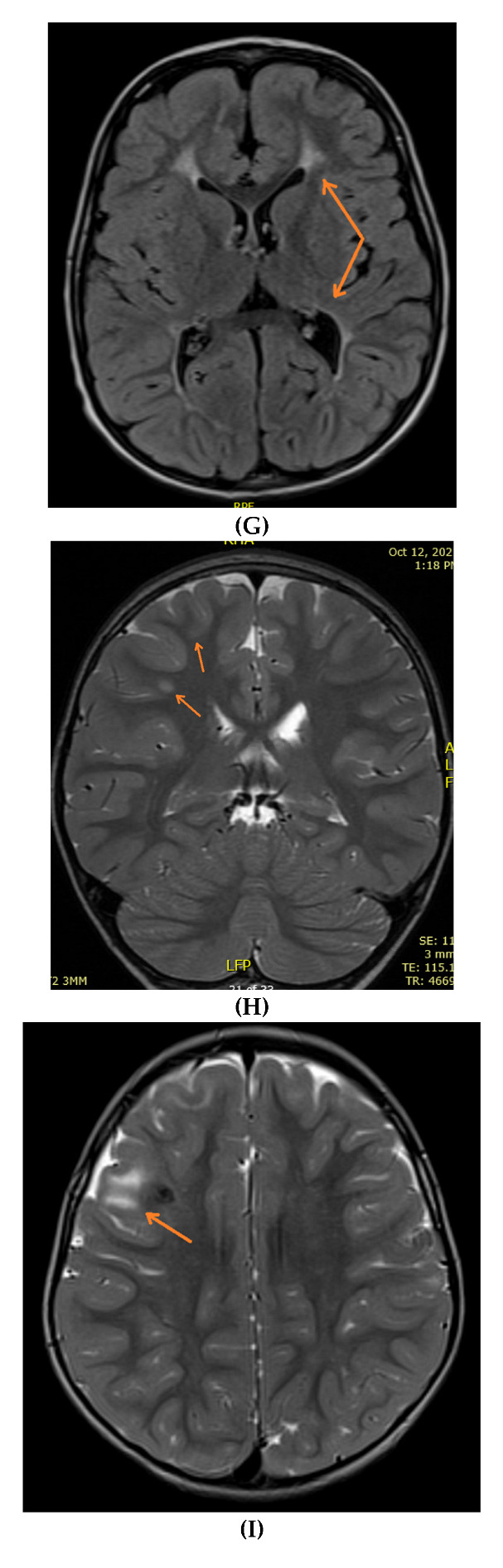

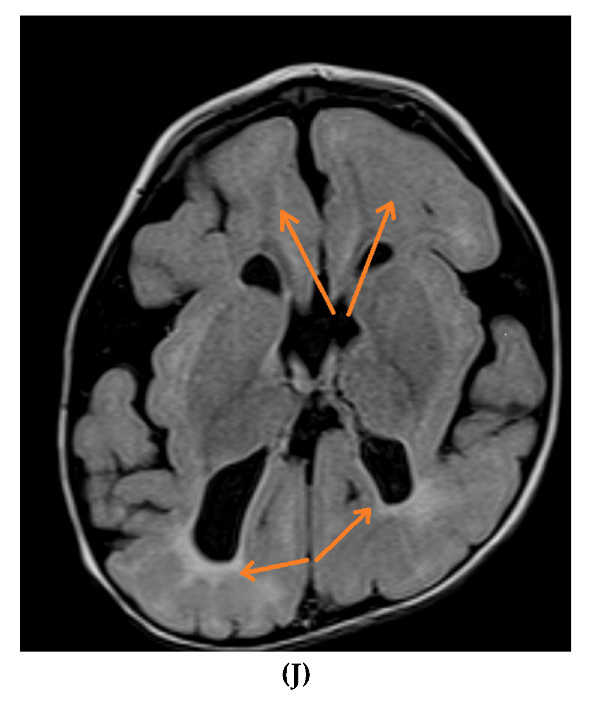
Brain MRIs showing the following (**A**): signs of hypoxia (T2 axial) with diffuse periventricular leukomalacia, enlarged ventricles and global atrophy (arrows); (**B**): delayed myelination in a 2-year-old child with diffuse hyperintense white matter compared to gray matter and brainstem (T2 axial); (**C**): dysgenesis of corpus callosum affecting its rostrum, genu, trunk and splenium (T1 sagittal) (arrows); (**D**): diffuse brain atrophy with enlarged ventricles (T1 axial); (**E**): severe ventricular dilatation (T2 axial) in a child with congenital muscular dystrophy; (**F**): intraventricular hemorrhage III (arrows) and hydrocephalus (T1 axial); (**G**): periventricular leukomalacia (T2 axial) (arrows); (**H**): non-specific white matter changes right precentral (T2 coronal) (arrows); (**I**): right frontal cortical tuber in a child diagnosed with tuberous sclerosis (T2 axial) (arrows); (**J**): PVL and frontal pachygyria (T2 axial) (arrows).

There were 32 female patients (57%) and 24 male patients (43%) (Table S1). The mean age for seizure onset was 5.9 ± 2.7 months, and the age range was from 2 to 13 months. Forty-one patients (73%) had active epilepsy, and the remaining fifteen patients had seizures under clinical control. About half of the cohort was diagnosed with global developmental delay (51%). The majority of the families were consanguineous (59%) (Table 1). Hypoxic ischemic encephalopathy was seen in ten MRIs (17%); nine MRIs (16%) were normal; nine MRIs (16%) showed delayed myelination; eight MRIs (14%) showed thinning and dysgenesis of the corpus callosum; seven MRIs (13%) showed the dilatation of ventricles, atrophy and hydrocephalus; five MRIs (9%) showed congenital migration disorders; and the remaining MRIs (15%) showed periventricular leukomalacia, cortical tubers, non-specific white matter changes, basal ganglia abnormalities and the consequence of meningitis (Figure 1 and Figure 2). Death occurred in three patients (5%).

Out of the 56 MRIs carried out initially, 28 MRIs were abnormal (50%). Upon follow-up, 47 MRIs (84%) were reported as abnormal, which was statistically significant (*p* < 0.05). Most of the children with active epilepsy and developmental delay had abnormal second MRI brain scans (64% and 82%, respectively) (Table 2). The second MRI brain scans in children with active epilepsy and developmental delay also showed statistically significant values (*p* = 0.05) (Table 3). 

## 4. Discussion

In this study, brain MRI findings in children diagnosed with infantile spasms were reviewed. As part of the etiological work-up of infantile spasms, brain MRI is of utmost importance. The yield of MRI brain scans can reach up to 75% [17,18,19]. One of the common etiologies of infantile spasms is asphyxia. Asphyxia can be detected in 18–80% of children with infantile spasms [20,21]. In this cohort, hypoxia occurred in ten patients (17%). 

The consequences of intrauterine infection and meningitis were also reported. Cerebrovascular causes, such as ischemic stroke, represent around 5–10% of reported etiologies. Malformations of cortical development (MCD), such as lissencephaly, heterotopias, polymicrogyria and focal cortical dysplasia, can be detected in 8–20% of cases [22,23]. 

Infantile spasms’ etiologies related to delayed myelination and MCD should be considered, even if normal MRI results are found in young infants, and a repeat MRI brain scan is recommended, especially after the age of 2 years. In this study, 20 (35%) out of 56 patients initially had normal MRI brain scans. In general, reviewing MRI brain scans in infants can be challenging due to progressive age-related myelination. Myelination patterns differ monthly in the first 2 years of life. Findings such as brain atrophy, dilated ventricles, MCD, white matter diseases, sequalae of infections and asphyxia might not be detected in infants’ MRIs. The brain myelination process correlates very closely to developmental milestones. It occurs in central to peripheral, caudal to rostral and dorsal to ventral patterns. Sensory regions develop followed by motor areas [24,25]. 

MRI brain scans are superior to CT scans for the evaluation of the myelination process. Unmyelinated white matter is hypodense on CT scans. In the first year, myelination can be seen as an MRI T1 sequence hyperintensity. T2 is the most sensitive sequence in children aged 1–2 years old, demonstrating a gradual shift from hyper- to hypo-intense changes. 

However, in resource-limited institutes, CT scans can be helpful for the evaluation of few IS-related etiologies, such as intrauterine infections, calcifications and cerebral atrophy [26,27].

A positron emission tomography (PET) scan is another imaging modality that can be utilized in the evaluation of IS. In intractable IS with negative MRI findings, PET scans can increase the yield of finding focal abnormalities by up to 20% [28,29].

The outcome of infantile spasms is usually poor. In this study, 73% of patients continued to have epilepsy. In general, epileptic encephalopathies and intractable epilepsy, such as Lennox–Gastaut syndrome, were observed in 60–85% of patients after IS. About 90% of infants with IS in this study had developmental issues, such as autism spectrum disorder, speech delay, intellectual delay and global developmental delay. Similar to other studies, developmental disorders were common in this condition. Five percent of this cohort unfortunately died. Premature death in infantile spasms was reported to be between 5 and 30% in different studies [30,31]. Another observation was the high percentage of consanguinity in this cohort (59%), which is similar to the reported consanguinity numbers of 52.1–67.7% in Saudi Arabia [32].

Active epilepsy and developmental delay were observed in children with abnormal MRI brain scans in this study. Injury to the developing brain, especially in the first two years of life, can affect neurotransmitters’ function and neurophysiological integrity. It could also lead to neocortical excitability with increased susceptibility to seizure generation [33,34].

## 5. Conclusions

Using the ILAE Commission on the Classification and Terminology’s recommendation to classify IS according to etiologies such as asphyxia and genetic, structural, metabolic and unknown etiologies will be clinically helpful to better understand and treat IS.

For IS investigations, MRI brain scanning remains an important diagnostic tool. Repeating MRI brain scanning should be considered, especially if the initial MRI brain scan is normal. Various structural etiologies of MRI brain scans are associated with future active epilepsy and developmental delay. A higher percentage of active epilepsy and developmental concerns can be seen in children with abnormal MRI brain scans and infantile spasms.

## Figures and Tables

**Table 1:** Summary of clinical characteristics of the cohort.

Variables	Variables	Number (n)	%
Gender	Female	32	57.1%
	Male	24	42.9%
Seizure outcome	Active epilepsy	41	73.2%
	Controlled	15	26.8%
Development outcome	Normal	5	8.9%
	GDD	29	51.8%
	Intellectual delay	11	19.6%
	Speech delay	8	14.3%
	ADHD	1	1.8%
	ADHD/ASD	1	1.8%
	ASD	1	1.8%
Clinical diagnosis of seizure types	Generalized	28	50%
	Focal	13	23.2%
	LGS	15	26.8%
Consanguinity	No	23	41.1%
	Yes	33	58.9%
Similar cases in the family	No	54	96.4%
	Yes	2	3.6%

**Table 2:** Seizure outcome, ASM, GDD and MRI brain relation.

Variable	Groups of Study	2nd MRI Normal	2nd MRI Abnormal
		No (%)	No (%)
Seizure Outcome	Controlled	4 (7.1%)	11 (19.6%)
	active epilepsy	5 (8.9%)	36 (64.3%)
Current ASM	current ASM (≤2)	4 (7.1%)	29 (51.8%)
	current ASM (3 and more)	5 (8.9%)	18 (32.1%)
Development Outcome	Normal	4 (7.1%)	1 (1.8%)
	Developmental delay	5 (8.9%)	46 (82.1%)

ASM: antiseizure medication.

**Table 3:** Seizure outcome, current AS and developmental outcome in relation to outcome and 2nd MRI brain follow up. Odds ratios in the detailed regression tables (logistic regression) between the 2nd abnormal MRI with the listed variables.

Variables	Groups of Study	Coef. Value	*p* Value	95% Conf. Interval	
Seizure Outcome	Controlled	0.962	0.202	0.087	1.675
	Active epilepsy	0.754	0.05	0.597	11.497
Current ASM	Current ASM (≤2)	0.720	0.335	0.335	1.547
	Current ASM (3 and more)	1.451	0.497	0.729	2.886
Development Outcome	Normal	8.567	0.998	1.00	1.00
	Developmental delay	0.497	0.05	0.650	0.380

## Data Availability

The data presented in this study are available on request from the corresponding author.

## Supplementary Materials

The following are available online at https://www.mdpi.com/article/10.3390/neurolint14010021/s1, Table S1: clinical characteristics of the cohort.
