# Supplementary material for: Brain Magnetic Resonance Imaging Findings in Infantile Spasms
Source: Neurol Int. 2022 Mar 3;14(1):261–70. doi: 10.3390/neurolint14010021 (PMC8952776; doi:10.3390/neurolint14010021)
Supplement: Supplementary file 1 [file neurolint-14-00021-s001.zip › neurolint-1563621-supplementary.pdf]

Table S1. Clinical characteristics of the cohort.

Patient	Gender	Age of seizures onset (months)	1st MRI brain	Age of 1st MRI (months)	2nd MRI brain	Age of 2nd MRI (years)	Seizure outcome	Current ASMs	Development outcome	Clinical diagnosis / Seizure type	Consanguinity	Similar cases in the family
1.	M	3 months	Normal	3	Normal	2	Controlled	3	GDD	LGS	Yes	Yes
2.	M	7	Normal	2	Normal	2	Controlled	2	Normal	Generalized	No	No
3.	M	2	Normal	1	Normal	2	Controlled	1	Normal	Generalized	No	No
4.	F	7	Normal	2	Normal	4	Active epilepsy	3	GDD	LGS	Yes	No
5.	F	6	Normal	2	Atrophy	3	Active epilepsy	2	GDD	Generalized	No	No
6.	M	13	Heterotopia	5	Heterotopia	2	Active epilepsy	2	GDD	LGS	Yes	No
7.	F	2	Dysgenesis of corpus callosum	3	Dysgenesis of corpus callosum	2	Active epilepsy	3	GDD	LGS	Yes	Yes
8.	F	12	Hydrocephalus	3	Hydrocephalus	2	Active epilepsy	2	GDD	Focal	Yes	No
9.	M	6	Hemimegalencephaly	2	Hemimegalencephaly	2	Active epilepsy	4	GDD	Focal	No	No
10	F	6	Hydrocephalus	2	Hydrocephalus	2	Active epilepsy	3	GDD	Focal	No	No
11	M	7	Cobblestone lissencephaly	1	cobblestone lissencephaly	3	Active epilepsy	4	GDD	LGS	Yes	No
12	M	8	Basal ganglia enhancement	5	Basal ganglia enhancement	2	Active epilepsy	1	GDD	Generalized	Yes	No

13.	F	4	Normal	4	Delayed myelination	3	Active epilepsy	2	GDD	Generalized	Yes	No
14.	F	7	Hydrocephalus	6	Hydrocephalus	2	Active epilepsy	2	ADHD	Generalized	No	No
15.	F	9	Normal	3	Delayed myelination	2	Active epilepsy	2	ASD	Generalized	Yes	No
16.	F	10	Thinning of the corpus callosum	2	Thinning of the corpus callosum	2	Active epilepsy	2	GDD	LGS	Yes	No
17.	M	4	Normal	4	PVL	2	Controlled	2	GDD	Focal	No	No
18.	M	4	Normal	3	PVL	2	Active epilepsy	3	GDD	Focal	No	No
19.	F	3	Normal	2	White matter changes	4	Active epilepsy	2	GDD	Generalized	Yes	No
20.	M	4	Normal	3	White matter changes	3	Active epilepsy	3	GDD	Generalized	Yes	No
21.	F	7	Delayed myelination	3	Delayed myelination	3	Active epilepsy	1	GDD	LGS	No	No
22.	F	6	Delayed myelination	3	Delayed myelination	2	Controlled	2	GDD	Focal	No	No
23.	F	4	Normal	2	Dilation of cerebral ventricles (atrophy)	4	Active epilepsy	2	GDD	Generalized	No	No
24.	M	8	Hypoxic ischemic encephalopathy	1	Hypoxic ischemic encephalopathy	2	Active epilepsy	3	GDD	LGS	No	No
25.	F	5	Normal	2	Heterotopia	2	Active epilepsy	3	GDD	LGS	Yes	No
26.	M	9	Hypoxic ischemic encephalopathy	3	Hypoxic ischemic encephalopathy	2	Controlled	2	GDD	LGS	Yes	No

27.	M	11	Dysgenesis of corpus callosum	3	Dysgenesis of corpus callosum	2	Active epilepsy	3	GDD	Generalized	Yes	No
28.	M	12	Normal	4	Hypoxic ischemic encephalopathy	3	Controlled	1	Speech delay	Generalized	Yes	No
29.	F	3	Delayed myelination	3	Delayed myelination	2	Controlled	2	Speech delay	LGS	Yes	No
30.	F	7	Thinning of the corpus callosum	3	Thinning of the corpus callosum	1	Active epilepsy	2	Intellectual delay	Focal	Yes	No
31.	M	6	Normal	3	Normal	2	Active epilepsy	3	Intellectual delay	Focal	Yes	No
32.	F	4	Dysgenesis of corpus callosum	4	Dysgenesis of corpus callosum	2	Controlled	2	Intellectual delay	Generalized	Yes	No
33.	F	5	Normal	5	Normal	2	Active epilepsy	3	Speech delay	Generalized	No	No
34.	F	4	Hypoxic ischemic encephalopathy	1	Hypoxic ischemic encephalopathy	3	Active epilepsy	3	Speech delay	Focal	Yes	No
35.	F	8	Hypoxic ischemic encephalopathy	1	Hypoxic ischemic encephalopathy	3	Controlled	1	Intellectual delay	Generalized	No	No
36.	M	3	Normal	2	Delayed myelination	2	Active epilepsy	2	Speech delay	Generalized	Yes	No
37.	F	10	Normal	2	Hypoxic ischemic encephalopathy	1	Active epilepsy	3	GDD	Generalized	No	No
38.	F	9	Normal	1	Thinning of the corpus callosum	2	Active epilepsy	3	GDD	Generalized	Yes	No
39.	F	6	Heterotopia	3	Heterotopia	2	Active epilepsy	3	GDD	Generalized	Yes	No
40.	M	4	Thinning of the corpus callosum	3	Thinning of the corpus callosum	3	Controlled	3	Intellectual delay	Generalized	No	No
41.	F	7	Normal	1	Normal	3	Active	3	Speech	Generali	Yes	No

							epilepsy		delay	zed		
42.	M	3	Normal	1	Normal	3	Active epilepsy	2	Normal	Focal	No	No
43.	M	5	Normal	2	Cortical tubers	2	Active epilepsy	2	Intellectual delay	Generalized	Yes	No
44.	F	3	Hypoxic ischemic encephalopathy	3	Hypoxic ischemic encephalopathy	3	Active epilepsy	2	GDD	LGS	No	No
45.	M	7	Normal	4	Meningitis sequelae	1	Controlled	2	Intellectual delay	LGS	Yes	No
46.	F	5	Dilation of cerebral ventricles	1	Dilation of cerebral ventricles	2	Active epilepsy	2	Intellectual delay	Generalized	Yes	No
47.	M	4	Hypoxic ischemic encephalopathy	2	Hypoxic ischemic encephalopathy	2	Active epilepsy	3	GDD	LGS	No	No
48.	F	4	Normal	5	Normal	2	Controlled	2	Normal	Generalized	Yes	No
49.	F	3	Normal	3	Cortical tubers	3	Active epilepsy	2	ADHD/ASD	Focal	No	No
50.	F	6	Normal	1	Dilation of cerebral ventricles	4	Controlled	2	Normal	Focal	Yes	No
51.	F	1	Delayed myelination	2	Delayed myelination	3	Active epilepsy	2	Intellectual delay	Generalized	Yes	No
52.	M	6	Thinning of the corpus callosum	4	Thinning of the corpus callosum	3	Controlled	2	Speech delay	Generalized	No	No
53.	M	8	Normal	1	Delayed myelination	4	Active epilepsy	3	Intellectual delay	Focal	Yes	No
54.	F	5	Normal	5	Hypoxic ischemic encephalopathy	2	Active epilepsy	3	Intellectual delay	LGS	Yes	No
55.	F	4	Delayed	3	Delayed	2	Active	2	GDD	Generali	No	No

			myelination		myelination		epilepsy			zed		
56.	M	3	Normal	2	Hypoxic ischemic encephalopathy	2	Active epilepsy	3	Speech delay	Generali zed	No	No

LGS: Lennox-Gastaut syndrome

ASM: antiseizure medications

GDD: global developmental delay.

PVL: periventricular leukomalacia

ADHD: Attention deficit hyperactivity disorder

ASD: autism spectrum disorder
